# Biochemical profile changes in stored donor blood for transfusion

**DOI:** 10.12669/pjms.35.6.220

**Published:** 2019

**Authors:** May AlMoshary, Eman Al Mussaed, Maria Arab-din

**Affiliations:** 1 Dr. May AlMoshary. MD. Basic Science department (Hematology), College of medicine, Princess Nourah Bint Abdul Rahman University, Riyadh, Saudi Arabia; 2 Dr. Eman Al Mussaed. MD. Basic Science department (Hematology), College of medicine, Princess Nourah Bint Abdul Rahman University, Riyadh, Saudi Arabia; 3 Dr. Maria Arab-din. MBBS, MPhil (Scholar). Khyber Medical University, Peshawar, Pakistan

**Keywords:** Storage lesion, Blood transfusion, Hyperkalaemia

## Abstract

**Objectives:**

This study’s aim was to find out that how various biochemical parameters of donor blood are affected during storage.

**Methods:**

This cross-sectional study was conducted in hematology Unit of Rehman Medical Institute, Peshawar and Fatimid Foundation, Peshawar Khyber Pakhtunkhwa, over a period of six months from June 2018 to November 2018. This study includes 300 healthy volunteer donors. Analysis of stored blood was done at 0, 3, 7, 14- and 21-days interval. Data were recorded and analyzed in SPSS v 20. A p value of less than 0.05 was taken as significant.

**Results:**

Three hundred healthy volunteer donors were included in the study in which 63% were male and 37% were females. Mean age was 26.54±7.3 years with age range from 23-46 years. Out of 300 donors 15.33% were O+, 35.33%, 9.66% B+, 8.33% A+, 7.66% AB+, 7.66% O-, 7% A- and 9% B-. Significant. Changes were observed in serum potassium, LDH, pH, serum chloride, serum sodium and AST levels. (p:<0.001). however, storage did not affect rest of the parameters.

**Conclusion:**

This study reveals that during storage certain changes occur in haematological and biochemical parameters which ultimately may put patients at risk.

## INTRODUCTION

Annually almost 11 million units of red cells transfusion occur, making it one of the most common medical intervention.^1^ Ordinarily red cells are administered as a concentrate, known as packed cells with preservative that can be refrigerated for almost 42 days. In order to treat various medical diseases blood transfusion with allogenic blood products is a common medical intervention,^2^ such as anaemia resulting from infection, disorders related to blood synthesis, drugs related cytotoxicity and blood loss in accident, during surgery.^3^ So to improve oxygen delivery to tissue and to lessen the complication of anaemia or related medical procedures transfusion is intended.

All over the world almost 107 million units of whole blood were collected in 2013 of which 67% of blood transfusions in low income countries were administered to children below five years of age where as patients above 65 years constitute relatively 76% of transfusion in high income countries.^4^

The most common transfused blood component in developed countries are red cells. In contempt of presages that red cells use could increases as population ages with greater use of transfusion in suffering from cancer or cardiac disease, the number of red cell transfusions has decreased from high of approximately 15 million units in 2008 to almost 12 million unit in 2015. Over time red cells transfusion have decreased from 50/1000 population to 40/1000.^5^

Appropriate preservation and long-term storage of RBCs is required in order to assure promptly obtainable safe supply for transfusion medicine. RBCs can be stored up to 42 days as per the food and drug administration norms, the storage lesion is a denomination that typically comprises all changes which occurs as RBCs age while in storage solution. These changes lead to haemolysis with concomitant rise in extracellular free iron, haem and haemoglobin that results in nitric oxide diminished bioactivity because of scavenging structural alteration, lactic acid and calcium/potassium build-up, a decline in 2-3 DPG and ATP, deduction in PH and glycolysis rate, and an accumulation of shed bioactive proteins, lipids.^6^-^8^Serious complications and death in severely ill patients, especially in those patients who are to be operated for any cardiac disease who have received immense transfusion.^9^

Our objective was to find the changes in biochemical parameters in whole blood stored in the blood bank in a resource-limited setting. The findings of this study will provide us with local data for further research.

## METHODS

The present study was conducted in Haematology department, Rehman Medical Institute, Peshawar, Fatimid Foundation, and Peshawar Khyber Pakhtunkhwa from June 2018 to November 2018. From 300 healthy volunteer donors, 450 ml of blood was drawn into citrate phosphate dextrose adenine (CPDA) bags. With due safety precautions blood was collected to avoid contamination and infection. All subjects were serologically tested for hepatitis C, B, and HIV virus. In an isolation shelf in the blood bank at 2-4 C^o^ blood bags were precisely stored. Out of each blood bag a 25ml of blood was taken for the sake of study and was stowed in plain bags. Analysis of stored blood was done at 0, 3, 7, 14- and 28-days interval by withdrawing 5ml blood each time from the bag. The sample evaluated on day 0 served as control.

By using standard ranges of various parameters used in this study are as follows: urea (10-50 mg/d/L), creatinine (0.7-1.3 mg/dL), ionized calcium (1.1-1.35 mmol/L), AST (upto 40 U/L), ALT (upto 40 U/L), total proteins (6-8 g/L), LDH (235-470 U/L), sodium (135-155 mEq/L), potassium (3.5-5.5 mEq/L), chloride (96-112 mEq/L) and pH (7.35-7.45). The study was approved by the Ethics Committee of the Rehman Medical Institute, Peshawar Pakistan. (DIR/KMU-EB/CB/000603 dated June 9, 2018)

### Statistical Analysis

Standard statistical approaches were used to find the mean and standard deviation. Chi square test was used to find the effect of blood storing on its hematochemical parameters. A p value less than 0.05 was considered as significant. The control used is blood at zero time. All values were quoted as the mean±SD.

## RESULTS

Out of 300 donors, 189(63%) were male and 111(37%) were females. Mean age was 26.54±7.3 years with age range from 23-46 years. Out of 300 donors 46(15.33%) were O+, 106(35.33%) 29(9.66%) B+, 25(8.33%) A+, 23(7.66%) AB+, 23(7.66%) O-, 21(7%) A- and 27(9%) B -. Numerous biochemical parameters are shown in Table-I. Noteworthy changes were observed in serum sodium, potassium, chloride, AST, LDH, and PH, (p<0.001)). On the other hand, the other parameters were not affected by storage. A steady increase in potassium values were observed.

**Table I:** Showing biochemical parameters at day 0, 3, 7, 14 and 28 of storage.

Parameters	Day 0	Day 3	Day 7	Day 14	Day 28	P value
Sodium	152.8 ± 4.01	150.1 ± 2.89	147.9 ± 1.41	143.1 ± 1.97	141.9± 3.99	<0.001
Potassium	4.33±1.29	6.73±2.43	9.93±2.97	14.16±4.56	19.89±4.01	<0.001
Chloride	86.32±1.96	89.55±2.05	93.91±2.44	96.83±2.19	91.34±1.09	<0.001
Calcium	0.06+0.007	0.062±0.005	0.063±0.004	0.0067±0.001	0.0066±0.021	NS
Urea	27.71±3.99	25.19±2.70	26.11±3.18	24.32±2.45	24.17±2.56	NS
Creatinine	0.99±0.04	1.02±0.02	1.07±0.04	1.01±0.06	1.02±0.01	NS
AST (mg/dl)	21.95 ± 4.91	23.54±6.32	28.43±3.22	38.26±9.90	44.31±8.55	<0.001
ALT (mg/dl)	40.65±13.65	40.43±18.89	39.54±23.66	44.87±13.76	46.32±10.87	0.487
LDH (mg/dl)	202.54±17.87	289.21±23.98	487.91±97.93	523.65±113.54	643.32±187.8	<0.001
Proteins (g/dl)	6.76±0.77	6.43±0.76	5.99±0.11	6.87±0.3	6.7±0.88	NS
PH	7.22±0.18	7.01±0.33	6.91±0.44	6.89±0.23	6.77±0.54	<0.001

We also stratified the blood group with the clinical parameters like potassium, chloride, AST, LDH and PH and we observed that different blood groups had no effects on clinical parameters and p-value was found insignificant.

## DISCUSSION

Despite advantages of blood transfusion, which is an effective method to avert anemia, preparing patients for surgical interventions and reduce symptoms of blood loss, it may be threatening to recipients. Meanwhile storage of whole blood in the blood bank, various alteration was noticed. A few parameters showed elevated where as some revealed reduced values.

Results of this study demonstrate many potential noxious changes that occur in the blood during storage. An exchange of content between plasma and red cells occurs because of prolong contact of plasma with RBCs that leads to changes in analyte concentrations as well as dilution. Potassium levels marked up within three days and this increase persist subsequently. from day 1 to day 28 of storage there was a notable ascent in K concentration ((<0.001), almost 1 mEq/L of the extracellular K level increase daily in stored blood, which revealed that there is higher concentration during the early days of storage.^10^

One of the study from Ghana demonstrated that there is increase in concentration of potassium on day 10^th^ and 15^th^ by 21.4% and 60.7% respectively.^11^ An upsurge in ECF potassium concentration decreases in ECF sodium concentration and will result in collateral opposite effect in ICF where NA and K+ concentrating changes as well because of failure of Na-K pump. Wallas first hypothesized the failure of Na-K ATPase action during the storage period, under normal condition Na-K pumps 3 Na+ ions out of the cells for 2 K+ ions pumped in.^12^ suppression of pump cause hyponatremia and hyperkalaemia as to be found in the present study. A similar study by Jobes et al.^13^ showed the same finding regarding sodium as in this study, where declining level of sodium were reported during the storage of blood.

Initially up to seven days chloride level accelerated after which a notable decline was observed. Sodium, calcium, and chloride are low molecular compounds and by storing, these enter into RBC’s under the effect of their concentration gradients.^14^

In AST level considerable change (p<0.001) were noticed which could be due to haemolysis as observed by other authors. Koseoglu et al. study showed that haemolysis interference affected LDH and AST almost at untraceable haemolysis by visual inspection (plasma haemoglobin <0.5 g/L) while clinically expressive differences of potassium and total bilirubin were observed in moderately haemolysed samples (haemoglobin >1 g/L). ALT, cholesterol, gamma-glutamyltransferase (GGT), and inorganic phosphate concentrations were not interfered up to severely haemolysed levels (haemoglobin: 2.5-4.5 g/L).^15^

In the present study it was also found that PH of stored blood decline during storage, also the drop in PH elevated with increase in storage time. (*p*=0.001). The drop in the PH would be due to upsurge in the level of lactate because of anaerobic metabolism of glucose and besides the drop in PH was directly proportional to the surge in lactate level.^16^ On 0-day pH was within normal range which fell to 6.77 at the end of 28th day. For every 0.1 unit of pH alteration results in a 0.4 mmol/L alteration in the serum potassium level. Acidosis cause rise in Potassium levels while alkalosis reduced the potassium level.^17^ It has been shown that a decline in pH and levels of lactate and potassium start rising within a couple of hours of storage while other changes may take weeks to appear.^8^

## CONCLUSION

This study showed that variations to several hematological and biochemical parameters occur during the storage of blood, despite storing blood in CPDA bag, and subsequently may cause untoward risks to patients. It is better to give patients fresh blood which has been stored for less than seven days.

### Authors’ Contribution:

**MAM, EAM:** Conceived, designed and did statistical analysis & editing of manuscript.

**MA:** Did data collection and manuscript writing.

**MA, MAM, EAM:** Did review and final approval of manuscript.
